# The gut-microbiome-endocannabinoid axis and anhedonia/amotivation: A mediation analysis in a general population cohort

**DOI:** 10.1192/j.eurpsy.2021.365

**Published:** 2021-08-13

**Authors:** A. Minichino, M. Jackson, P. Burnet, B. Lennox

**Affiliations:** Psychiatry, University of Oxford, Oxford, United Kingdom

**Keywords:** Microbiome, Cannabis, negative symptoms, mediation

## Abstract

**Introduction:**

General-population studies investigating the biological correlates of anhedonia/amotivation might be informative for treatment breakthroughs for a number of clinical conditions. Reduced gut-microbial diversity might lead to an anhedonic/amotivational syndrome (“sickness behaviour”). However, how gut-microbial diversity contribute to this clinical phenotype is a key gap in knowledge. We hypothesised the endocannabinoid system would be at play.

**Objectives:**

We tested the hypothesis that the endocannabinoid system mediates the association between gut-microbial diversity and anhedonia/amotivation

**Methods:**

Secondary data analysis on 786 volunteer twins (TwinsUK). Measures of gut-microbiome, faecal endocannabinoid metabolites, and anhedonia/amotivation were collected over five years. To test our hypothesis we used a multilevel mediation model using alpha diversity as predictor, faecal levels of the endocannabinoid palmitoylethanolamide (PEA) as mediator, and anhedonia/amotivation as outcome. Analyses were adjusted for obesity, diet, antidepressants, and sociodemographic covariates.

**Results:**

Mean age was 65.2±7.6; 27% were obese and 4.7% were on antidepressants. Alpha diversity was significantly associated with anhedonia/amotivation (β=-0.37; 95%CI: -0.71 to -0.03; P=0.03). Faecal PEA levels mediated this association: the indirect effect was significant (β=-0.13; 95%CI: -0.24 to -0.01; P=0.03), as was the total effect (β=-0.38; 95%CI: -0.72 to -0.04; P=0.03). The direct effect of alpha diversity on anhedonia/amotivation was attenuated fully

**Conclusions:**

We provided the first evidence showing that the association between gut-microbial features and anhedonia/amotivation is mediated by the endocannabinoid system. These findings shed light on a new therapeutic target in an area of unmet clinical need.

**Disclosure:**

No significant relationships.

